# Ræstur fiskur: air-dried fermented fish the Faroese way

**DOI:** 10.1186/s13002-015-0064-9

**Published:** 2015-11-04

**Authors:** Ingvar Svanberg

**Affiliations:** Uppsala Centre for Russian and Eurasian Research, Uppsala University, Box 514, SE-751 20 Uppsala, Sweden

**Keywords:** Ethnozoology, Fermentation, Local fish knowledge, Ethnoichtyology, Traditional cuisine, Ethnogastronomy

## Abstract

**Background:**

Fish has played an important role in the diet of the population of the mid-Atlantic Faroe Islands. Dried and fermented fish in particular have been an essential storable protein source in an economy where weather conditions and seasonal fluctuations affect the availability of food. For generations the islanders have prepared *ræstur fiskur*, a home-made air-dried and fermented fish dish made of Atlantic cod (*Gadus morhua* L.) or saithe (*Pollachius virens* (L.)). Fermenting the fish is an efficient and valuable cultural strategy for preserving fish.

**Methods:**

This ethnobiological study investigates the historical and present use of fermented fish in Faroese cuisine and examines its preservation as an everyday food that Faroese men pride themselves on making in high quality. This study is based on field notes collected through interviews and observations on the Faroe Islands since the mid-1990s.

**Results:**

Processed fish could be stored for a long period of time; this was important in an economy where weather conditions and seasonal fluctuations affect food availability dramatically. For this reason, home-made air-dried fish has been central to the food security of the Faroese people. Usually consumed with tallow from sheep, the dish was once appreciated customarily on Christmas Eve and New Year’s Eve, but has been largely replaced by Danish dishes. However, it has survived as everyday food until today.

**Conclusion:**

The presence of small-scale fishing, changing economic conditions, socially acquired taste-preferences, and the importance of old-fashioned dishes as key symbols of cultural identity, all contribute to the survival of *ræstur fiskur* in Faroese food culture. Today, the dish is not only an essential food source, but its consumption is also an important act of identification and solidarity with the national identity of the islanders.

## Background

“The Faroe islander is very conservative in certain areas. He sticks to old way of professional practices […], old-fashioned costumes and archaic food habits” [1].

**Fig. 1:**
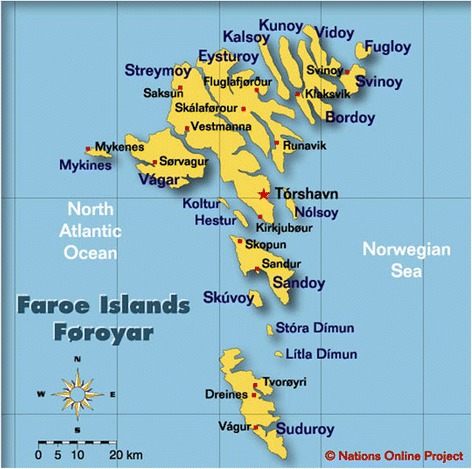
The Faroe Islands are an island group consisting of 18 major islands about 655 km off the coast of Northern Europe, between the Norwegian Sea and the North Atlantic Ocean, about halfway between Iceland and Norway. The closest neighbours are the Shetland Isles and the Outer Hebrides (Courtesy Nations Online Project)

Fish has always played an important role in the diet and culture of the local population of the Faroe Islands (Føroyar). The Faroes are located in the north-eastern Atlantic Ocean between Scotland and Iceland. The archipelago consists of 17 populated (out of 18) islands with a total area of 1,400 km^2^ and a population of close to 48,700 (1 Jan. 2015). Over the generations the ecological conditions and the relative isolation of the Faroe Islands has reinforced a strong cultural identity and its reliance on local foods, including fish. This strong connection between cultural identity and fishing, and indeed the island’s food security, continues to be expressed today. Few other countries today have such a high level of dependency on the sea and its fish resources as the Faroe Islands [2] (Fig. 1).

Since 1948, the islands have been a self-governing (home-rule) overseas administrative division of Denmark. In contrary to Denmark, the Faroe Islands refused to join the European Communities in the 1970s, mainly over the issue of fishing limits, which remains a contested subject. Today, the economy depends almost entirely on exports of fish and fish products, which in 2012 accounted for 91 % of exports by value and made up 20 % of the GDP.

This high dependence on fishing (and aquaculture) means the economy remains extremely vulnerable. In August 2013, the European Commission banned the import of the Atlanto-Scandian herring (*Clupea harengus* L.) and Atlantic mackerel (*Scomber scombrus* L.) from the Faroe Islands to the European Union [3]. Fishing therefore has economic, nutritional, and political dimensions.

Nowadays most Faroese people eat domestic duck (or sometimes locally farmed goose) with both boiled and caramelized potatoes (*brúnkað epli*) at Christmas (the pre-boiled, peeled potatoes are gently stirred in melted sugar with margarine). It is a Danish holiday dish introduced into the Faroese cuisine quite recently (during the 1960s and later). However, the time-honoured dish for Christmas Eve is air-dried fermented cod, so called *ræstur fiskur*, served with *sperðil* (a kind of very simple sausage made of sheep tallow and salt put in a casing of intestines). This ancient and very simple food habit, known as *aftanfiskur* (‘fish for the holiday Eve’), in connection with Christmas and New Year’s Eve, was still common forty years ago. It is rarely observed today, and only the older generations, especially in the north, retain this holiday tradition [4, 5].

**Fig. 2:**
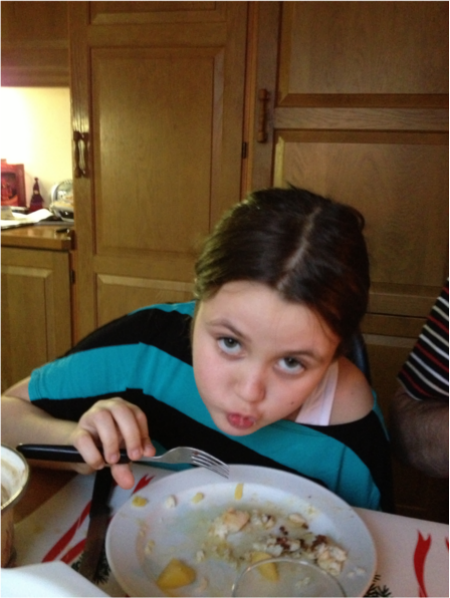
A Faroese girl enjoying a meal of *ræstur* fish with *garnatálg* (made of tallow from around the left colon) and potatoes in Vestmanna in December 2012 (Photo Osva Olsen)

While it has largely disappeared as a holiday dish, *ræstur fiskur* continues to be a popular everyday food among many Faroese people. Although many islanders still appreciate its unique taste, it is regarded by Danes and others as possessing a sharp, ripe, almost repugnant taste. Some foreigners learn to appreciate it; others live there for years and refuse to eat it. For the native population it is a taste acquired during childhood. To illustrate, in a small survey I made in the late 1990s on Faroese children’s food, fermented fish was generally listed as their favourite, ranking higher than ‘foreign food’ (*útlendskur matur*) such as pizza and spaghetti. This may have changed with the rapidly increasing influences of fast food and the wider range of foodstuffs now available in the grocery stores. Nevertheless many young people still enjoy *ræstur fiskur*, which for them is tasty food (Fig. 2).

Faroe Islanders are not alone in appreciating and using fermented fish as food. It is a well-known product in many parts of the world and its local consumption entails a very ancient way of preparing fish. East and South-east Asian food cultures are renowned for such products, but fermented fish is prevalent also in the traditional cuisines in Africa, the Middle East, the Mediterranean region, Siberia and other circumpolar regions, as well as in the Americas [6–8]. In North-western Europe fermented fish has become primarily a kind of festive cuisine, connected with holidays, celebrations or other feasts [9, 10]. However, the Faroese *ræstur fiskur* served with *garnatálg* (kneaded tallow from around sheep’s rectum) is still more or less a main course for eating on weekdays, and it is not connected with haute-cuisine or festive celebrations. It is simple home-made food.

The goal of this study is to examine the historical and contemporary use of *ræstur fiskur*, including its production, preparation and place in the local and national culture of the Faroes. Although there are several studies on Faroese food habits and changes over time, the importance of fermented fish is underrepresented [2, 5, 11–13]. Furthermore, the growing scholarly literature on fermented fish among subsistence-based peoples ignores the presence of fermented fish in the Faroes, although other Nordic countries have been studied previously [14, 15]. Data for the present study is based on field notes collected through interviews and observations on the Faroe Islands since mid-1990s.

### Fermented fish in Scandinavia

As a highly perishable, proteinaceous food, fish must be prepared properly for storage to ensure it will last for later consumption. Drying, fermentation, salting and smoking are time-honoured, intricate methods of accomplishing this and thereby ensuring the nutritive needs of family households are properly met. Among these, drying and fermentation are the oldest methods of fish preservation [16, 17].

In Northern Europe, there are long traditions of fermenting animal products for human food. While lacto-fermented vegetable products have not played any significant roles in local food culture in the Nordic countries (in contrast to Central-Eastern Europe, see [18, 19]), fermented fish (as well as fermented milk and meat products) are historically widespread among the pre-industrial peasantry. This is especially the case in the northern part of the Scandinavian Peninsula, in the Faroes and in Iceland. Fermented animal products were commonly appreciated, with widespread tastes acquired early in childhood [9, 16].

Today fermented fish is rarely eaten in Scandinavia and is largely limited to a festive food eaten on special occasions. Examples of such fermented fish dishes are *surströmming* (made from Baltic herring) in Sweden and *rakefisk* (made from trout, char or whitefish) in Norway [9, 20]. Icelandic fermented Greenland shark, *kæstur hákarl*, has become a rather exclusive snack reserved for consumption during festive events especially. *Gravlax* (‘the buried salmon’), a raw salmon dish cured in salt, sugar and seasoned with dill, is appreciated all over Scandinavia today. However, contemporary *gravlax* is a modern non-fermented product, and has no resemblance with the old fashioned traditional fermented salmon, whitefish, cyprinids and other fishes which was originally buried in the earth, hence the survival name ‘buried salmon’ [20–22].

Among the Nordic countries, the Faroe Islands are the only location where fermented fish continues to be used as an everyday food. There, it is still primarily produced in the home, and usually as a combined effort of the husband and wife. The entire preparation process is itself elaborate, involving catching, cleaning, drying, and fermenting the fish. Consumption occurs within the household. However, it is nowadays possible to buy *ræstur fiskur* in grocery stores on the Faroe Islands. It can also be bought online and delivered to people’s front door.

## Methods

This study is based on my field notes collected through interviews and observations since the mid-1990s. It is part of a larger ethnobiological research programme, focused on the activity context between humans and other animal species from the Faroes. I prefer, in accordance with Balée [23], the expression *activity context* from the one-sided perspective implied by the terms plant and animal uses [24–27]. Wild animal taxa in the local cuisine have been discussed; although my research has centred specifically on ethnozoological issues, plant knowledge data have also been collected [28, 29]. Edible wild plants, however, played a minor role in the traditional diet (and even less nowadays) of the islanders [30].

I conducted unstructured interviews with households in Vestmanna, Gjógv and Tórshavn (the capital of the islands), and also took part in various activities as an observant participator [31]. Fishing and the consumption of fish are part of daily life on the Faroe Islands. I have therefore had many opportunities to observe and participate in the preparation of and consumption of semi-dried fermented fish. My field notes from these observations are my main sources. Supplemental information on the importance of fish as food was gathered from ethnographic reports, travelogues and local historic accounts [2, 5]. One important aspect is not discussed in this context though, that is the lactic acid bacteria and enzymes involved in the fermentation process. There is of course an interesting triangle of human-fish-microbe active here, but I have not found any relevant research on the microflora of the Faroese *raestur fiskur* so far. It is a topic for future research.

### Ethnographic setting

Culturally, the Faroe Islands comprise a small and relatively homogenous society [32]. Although recent archaeological evidence places human colonization of the Faroe Islands between AD 400–600, the current inhabitants are descendants of Viking Norsemen that arrived in the ninth century [33, 34]. Being part of Denmark from 1523, the population is mostly Lutheran, belonging to the Faroese Lutheran Church (80 %). The remainder belongs to the Open Plymouth Brethren (15 %) and various charismatic movements. It is a rather conservative Christian nation compared to other Nordic countries. The islanders have their own native language of Norse extraction, but most people are by necessity bilingual in both their mother tongue Faroese and in Danish, the language of the colonial power [32].

Shepherding, hunting for seabirds – and occasionally pilot whales and seals – and some barley cultivation were the main base of the economy since human settlement until early twentieth century [35, 36]. We know from many ethnographic studies that populations with traditional lifestyles have detailed knowledge of various species in their surroundings [37]. This is also true for the Faroe islanders. Fishing from land was primarily to supply food for individual households. Only landowning farmers had one or more boats. Fishing was a necessary complement to other economic activities. Pilot whales (*Globicephala melas* (Traill)), grey seal (*Halichoerus grypus* (Fabricius)), and sea-birds were also important sources of nutrition [5, 13]. The locals recognized very few fish species in the folk taxonomy. Among these, even fewer were recognized as food, including the various cod species (Gadidae), halibut, *Hippoglossus hippoglossus* (L.), and the now almost forgotten thornback ray, *Raja clavata* L. [38–40]. The saithe (*Pollachius virens* (L.)), which sometimes appeared in large shoals inshore, was of special importance, since many people could harvest them in great numbers from the shore [41, 42].

**Fig. 3:**
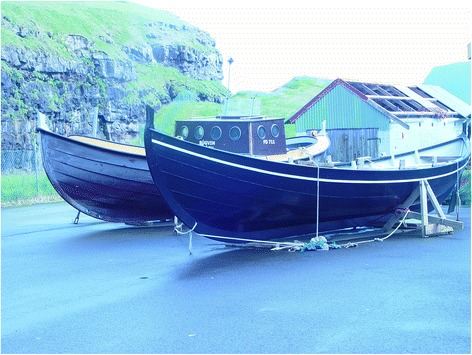
Traditional Faroese boats suitable for the Faroese waters, Gjógv, June 2004. These local rowing boats, which were previously used in many Faroese villages, have their origin in the smaller boats of the Vikings. Small open**-**boats are now mostly used for inshore fishing as a hobby (Photo Ingvar Svanberg)

During the twentieth century the economy of the Faroes transformed from small-scale coastal fishery to deep-sea fishery in the North Atlantic. Today, fishing is the primary economic activity [13, 43]. The Faroe Islands has a large fleet of trawlers and smaller line fishing boats, fish factories and also raises salmon (*Salmo salar* L.) and sea trout (*Salmo trutta trutta* L.) in fish-farms. Although most fish are harvested by these larger fishing vessels, some older men continue to fish for pleasure and for their own consumption in locally constructed boats, well adapted to the surrounding sea (Fig. 3). Due to rapid modernization, an increasing number of people are now found within the white-collar sector, or are engaged in service and other activities within the tertiary economic sector.

### Food preservation on the Faroe Islands

The island communities were up to the early twentieth century characterized by a storage economy [2, 5]. Food availability was seasonal with periods of predominately fresh foods, alternating with periods of mostly dried and fermented food. Up until the late 1960s and early 1970s, when home freezers became common, usual common way to preserve animal products on the Faroe Islands was by drying and fermenting. Mammals, birds and fish were wind-dried; no salt was used in the process [2, 11]. In 1846, Peter Ludwig Panum, a student in medicine and surgery, observed the food habits of the Faroe islanders. In addition to a kind of “gruel” (actually a soup) made of fat, small pieces of fermented dried meat and barley grain, Panum reports that they ate:

… *ræst*, that is, half-spoiled meat or fish. The same method of preserving meat which is used for lamb is used also for pilot whale meat, fish, or bird meat; all are hung up to dry without any preparation by salting, smoking, or the like. In the course of several months, when the meat (or fish) is neither fresh nor wind-dried, it is called *ræst*, a word that can be translated by no other term than ‘half rotten,’ an epithet fully merited by this meat, considering the abominable odour it spreads, its unpleasing, mouldy appearance, and it’s not infrequent occupation by maggots [44].

The Faroese politician Edward Mitens remembers from his childhood in the village Tvøroyri that most animal food products were hung up and eaten either as *ræst* or dried [45]. In the local cuisine some very old-fashioned dishes have survived up to the present day [46].

The results of a health and odontology survey conducted during the Great Depression in 1937–1938, indicated that the nutritional status of the otherwise poor Faroese villagers subsisting on traditional fish and meat diet was adequate from a health perspective [1]. This is further suggested by the fact that the birth weight at the time was higher than elsewhere in the Nordic countries [47].

Several of these traditional dishes are eaten by the islanders today [2]. For instance, the fermented and dry meat (always mutton) dishes *ræstkjøt* and *skerpikjøt* are especially highly valued. Also tallow in various forms, dried fish and dried meat are still widely-consumed old-fashioned Faroese dishes [2, 11].

### The importance of animal fat products

Animal fat is an important but seldom-discussed food source within the local Faroese cuisine [11]. Beside the small amount of butter that has been produced for local consumption in the Faroe Islands, the fat from sheep and the blubber from the pilot whale is widely used within the household as side dishes, not only for *ræstur* fish, but also for other dishes. Tallow (*tálg*) from sheep has played an important role in the local food culture. According to Faroese sheep-owners, several parts of the slaughtered sheep with good tallow can be used for various purposes; on greater and lesser omentum (the membrane surrounding visceral organs), kidney tallow, lump of tallow on the belly, tallow in the pelvis, heart tallow and the important tallow around the left colon [48]. Taking care of the tallow is generally considered woman’s work and those who possess this wisdom maintain intricate knowledge of the sheep’s anatomy. They know which kind of tallow is used to prepare various kinds of dishes. Suet is used when making fish balls (*knettir*, *frikadellur*), black pudding (*bloðmørur*) and other historically relevant customary foods.

**Fig. 4:**
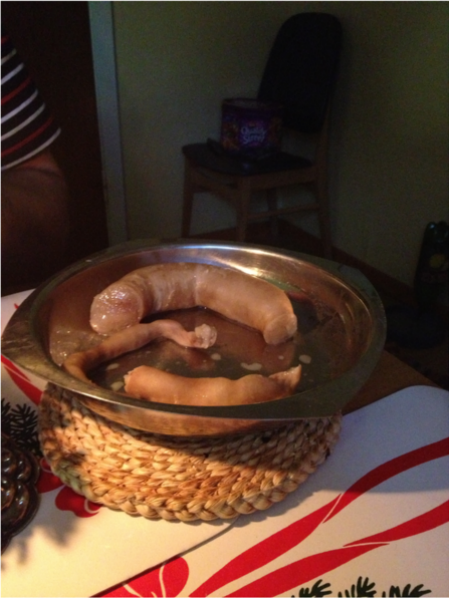
*Sperðil* is a local very simple sausage made of sheep tallow and salt put in a casing of intestines. Once a popular side dish for fish, whale meat and also as spread on bread; today it is becoming rare (Photo Osva Olsen)

*Sperðil* is a traditional dish made of the tallow around the sheep’s rectum, and prepared into a kind of primitive sausage [49]. It can be used as spread on bread, eaten with fish or added to the traditional unleavened bread (*drýlur*). In former times it was simply fried and eaten for breakfast (Fig. 4). Traditionally, when making *sperðil* people must keep quiet and the woman in charge was allowed only to use a protective magic rhyme during the process: *Sperðilin langi/av í kokuni gangi/ Sperðilin stutti/ av í kokuni prutti* ‘Rectum long/ now in the pelvis goes/ rectum short/ now in the pelvis spit’ [50].

*Garnatálg* is another archaic Faroese food stuff. This is made of tallow from around the left colon (*garnmørur*). Intestinal fat is formed into a large oval lump, which is air-dried. When preparing *garnatálg* it can be cut into slices and melted in a pan and served with *ræstur fiskur* [51].

*Spik*, or blubber obtained from the under hide of the pilot whale, is also an important food item. It is usually salted and consumed together with fish and whale meat. However, given to the high concentration of polychlorinated biphenyl (PCB) and mercury in the blubber, it is often replaced now with margarine [52]. However, many, of the older generation and the so-called “macho Faroese men”, continue to eat blubber [53].

### Processing and eating *raestur fiskur*

Making air-dried, fermented fish the Faroese way is a relatively uncomplicated process, although it requires intimate understanding of wind, temperature, and constituent micro-organisms and enzymes [17]. The air-drying process can be accomplished year-round, although summertime and early autumn are avoided because maggots (*maðkur*, lit. ‘worms’) often appear on the fish during that season. Larvae infestations are common in the humid, sultry weather in the autumn known as *maðkarveður* (‘maggot weather’) which is not considered suitable for making good air-dried fish.

**Fig. 5:**
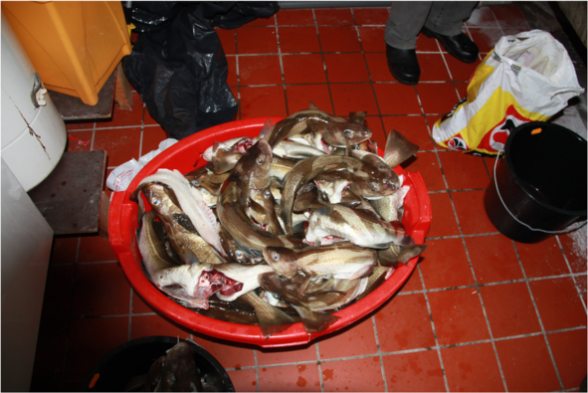
A day’s catch of small cods. Some will be eaten fresh or used in fish-cakes (*frikadellur*), others will be wind-dried outdoors to produce *ræstur fiskur*, July 2011 (Photo Osva Olsen)

Today, most fish used for making *ræstur fiskur* are caught by the man in the household from boats while at sea (Fig. 5). However, a few decades ago it was not uncommon for women and children to take part in fishing for saithe (*Pollachius virens* (L.)), with a rod standing on a rock, or *seiðaberg* (‘saithe cliff’), on the shore [4]. Today fishing from the shore is mostly only conducted by children. As in the past, the most commonly used fish for making *ræstur fiskur* is Atlantic cod, *Gadus morhua* L., saithe, *Pollachius virens* (L.), haddock, *Melanogrammus aeglefinus*) (L.), and occasionally ling, *Molva molva* (L.). The fish is beheaded and gutted before being hung up for the drying and fermenting process.

**Fig. 6:**
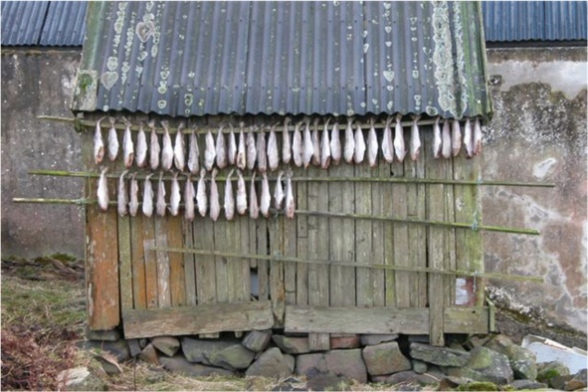
Traditional method of drying fish outside a *hjallur* or drying shed (Photo Erik Avnbøl 2008)

While meat (mutton) is hung indoors in the *hjallur*, a special kind of storehouse or shed with at least one, often two sides being made of laths, so that the wind can blow through and thus keeping it dry but cold (Fig. 6**)**, the fish is dried outdoors. For drying, the fish are tied together in pairs (*fiskagreipa*) at the tail and hung up under the roof outside. Small fish are best suited for air-drying. In addition, fish is sometimes dried on the fishing-boats, by hanging them to expose them to the sun and wind. When the fish starts to dry they are termed *visnaður*. Such *visnaður* fish (for instance saithe) can be eaten, but the skin should be removed before boiling it. After two to three weeks (depending on the weather conditions) they become *ræst,* but the difference between *visnaður* and *ræst* is fleeting. Later the fish are referred to as *turrur*, when it is fully dried. *Turrur fiskur*, which is rather expensive today, is eaten with fat (blubber or butter), either on its own as a snack, or with boiled potatoes as lunch or evening meal.

**Fig. 7:**
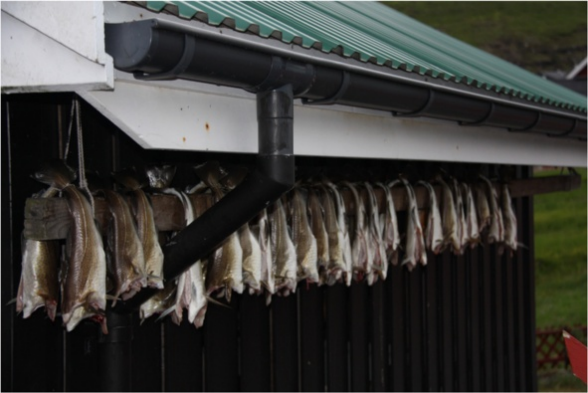
Air-drying outdoor under the roof of a modern house in Vestmanna, July 2011 (Photo Osva Olsen)

The steps deployed in the fermentation process are therefore important for making the fish taste good, and convey the breadth of culinary knowledge necessary for their continued procurement and consumption. If the weather turns humid during the drying process the fermentation will be interrupted, and the flesh of the fish will consequently spoil. For best results it is important to be observant, meticulous, and take good care of the fish while drying. Only the careful get preferable results. It is therefore necessary for the husband who is in charge of the drying to check the fish regularly, and if necessary move them to another spot under the roof (Fig. 7). In the past, when the fish was fully prepared, it was customarily stored in the *hjallur.* Today it is usually stored in the freezer.

Air-dried fermented fish is usually eaten cooked. The entire fish boiled together with the peeled off skin in salted water for 20 to 40 min. No other seasonings are added. When it is done, the skin and bones are removed on the plate and the fish is eaten with fat (*garnatálg*, *sperðil* or whale blubber) and boiled potatoes. The nutritional value of air-dried fermented cod is measured as energy per 100 g net: 622 kJ 36.4 g protein 0.09 g fat [54].

Up until a few decades ago, fermented cod head were also commonly eaten. This dish, called *grunnishøvd*, is made of cod heads which had been put in a bag with hay and kept in a cold room for three to four weeks. Although this dish has a particularly strong smell, it is cleaned and cooked accordingly for dinner [38, 55]. Although it was commonly eaten a few decades ago, it is increasingly difficult to find today.

In addition to serving as food for humans, wind-dried saithe was also used as supplementary winter fodder for cattle. It was valued as a protein-rich fodder during a season when grass was rare and the hay harvested from the small stripes of meadows were of lower quality [4].

## Discussion

**Fig. 8:**
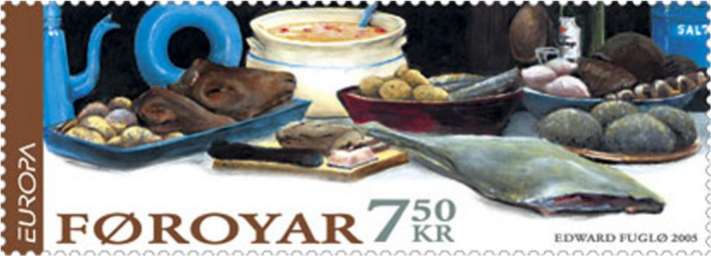
Faroese stamp with traditional foods. Artist Edward Fuglø, 2005

Contemporary Faroese community is a late-modern high-tech welfare state. As with other Nordic countries, the Faroese food culture is rapidly changing as new interests and tastes are introduced through many channels [53]. However, despite the influx of agro-industrial food products through convenience stores and supermarkets, many centuries-old dishes are still served. Dried and fermented mutton and several other local specialities are sought after rather expensive food, hard to get for the increasing urban population, but still eaten on holidays and festive occasions. They fit well on the buffet-style *kalt bord* (smorgasbord) usually served at festive occasions at rites of passage, such as Lutheran confirmation and wedding parties, both parties usually attended by large numbers of kinfolk, colleagues and neighbours [2]. This is especially true for traditional dishes such as fully dried fish, dry-salted whale blubber, cold cooked *ræstkjøt* and raw well-dried *skerpikjøt* (both made of air-dried mutton), all of which are served at large festive gatherings. Such traditional festive foods while expensive, are regarded as symbols for Faroese national culture, the “real” Faroese cuisine [56, 57]. These dishes were for instance depicted on postage stamps in 2007 (Fig. 8).

At this point, I return to the question posed at the beginning of the article: why has *ræstur fiskur*, unlike the festive fermented mutton dishes, remained an everyday food among the inhabitants on the Faroes? The everyday importance of *ræstur fiskur* can be explained by an interaction of several factors. First, unlike most other traditional foods, since *ræstur fiskur* is a simple dish that is easy to prepare within the household. It is a taste the islanders still acquired in early childhood. Recipes in modern Faroese cook books only suggest boiling it and eating with tallow or blubber [51, 55]. In addition, in contrast to locally produced mutton, which is expensive and rare (especially *skerpikjøt*), fish is readily available for most islanders.

The retention of *ræstur fiskur* as an everyday food is related to a larger cultural tendency towards conservatism in general, and with respect to food, specifically. This conservatism in food culture is likely generation-old, and is in part due to the geographical isolation of the islands. This is expressed by the fact that most Faroe islanders draw a line between what they regard as Faroese food (*føroyskur matur*) and imported food (*útlendskur matur* ‘foreign food’). As a result of this conservatism, Faroese cuisine today is relatively resistant to change, especially, compared to other Scandinavian cuisines, which typically readily integrate new ingredients and dishes.

The cultural conservatism of the Faroe islanders was further reinforced during World War II. At that time approximately 3,000 islanders that were forced to stay in Denmark during World War II, when Denmark was occupied by the Nazi-Germans and the Faroe Islands were occupied by British troops. When they were allowed to return to their islands in 1945 after five years away, traditional food gained increased popularity as a symbolic and tangible connection to their homeland and their cultural identity [58].

As a result of their history and geographic location, conservatism in everyday life and values are important traits in the Faroese community today. This is strongly expressed among the seamen. Many men spend months on commercial fishing vessels, and when returning home, they want the conditions to be “as it used to be”. Despite the fact that many of them have considerable expendable plenty incomes, they retain traditional values rooted in local village culture. As a result, they have a strong preference for local, traditional food [53].

Favouring and appreciation of local food has an important nationalistic dimension among many islanders. This is especially significant since the islands are still a colony of Denmark. Faroese nationalism affects many aspects of the society, including politics, language attitudes, world views and of course food ways [53].

Fermented fish and meat, together with pilot whale meat, have become key symbols among the Faroes [59, 60]. They have become symbolic for Faroese culture and preparing and eating these foods, reinforce Faroese cultural identity. As a result, these foods continue to be widely available, through a variety of avenues. For islanders who do not have the opportunity to fish themselves, or do not have access to kin who fish, they can buy locally produced air-dried fish at the local market in the harbour of Tórshavn and at supermarkets.

In the face of increasing globalization, the role of *ræstur fiskur* and other traditional foods in the Faroe Islands is changing. Today, the white-collar sector of an increasingly educated and modern urban middle-class has a growing interest in gourmet and restaurant food. This is especially true in Tórshavn. There is now a vogue for gourmet foods made exclusively from local ingredients, thus following the general European trend [61, 62]. For example, the exclusive avant-garde restaurant Koks in Tórshavn, with its chef Leif Sørensen, has made local food its trademark; they have also produced a cookbook. Fermented meat and fish is difficult to render in various forms, but it is obviously in their interest to integrate it into modern food as well.

As in other marginal areas of Scandinavia, the global Slow Food movement has inspired local interest among Faroe islanders in developing local food ways. For instance, the Outer Islands Association (Útoyggjafelagið), whose members come from eight small outer islands, joined the Slow Food movement in 2009 in an effort to promote local food products. They focus especially on local dishes made of mutton, but also include locally available food plants: garden angelica, *Angelica archangelica* L. (for making jam), rhubarb, *Rheum rhabarbarum* L. (for making jam, the Faroese rhubarb plants are considered low in oxalic acid), meadow sweet, *Filipendula ulmaria* (L.) Maxim. (using flowers for making cordial), and seaweed salt. In addition, some seafood from Faroese waters has also been promoted, especially Norway lobster (*Nephrops norvegicus* L.), which has a well-founded reputation for being high quality [63]. It is interesting that the fermented fish (or even the meat) is ignored by the representatives for the local Slow Food movement [64].

## Conclusions

The systematic study of local food production, consumption, and conservation is clearly central to many ethnobiological research projects [65]. By examining time-tested food ways such as those included here, scholars and others interested in food security issues can benefit by appreciating the knowledge, skills, and techniques and all associated cultural strategies necessary for maximizing subsistence. Understanding historic food preparation systems can also serve to advance food security policies in meaningful ways worldwide [62].

While local knowledge of plants and larger animals is disappearing in most parts of Europe, the activity context between fish and humans remains based on the efficiency of folk knowhow in many rural areas. Despite this, folk ichthyology in general, and fish as food in particular, are still a relatively understudied topical components of ethnobiology [65–67]. The process of making air-dried, fermented fish is one of many example of a resilient culinary tradition capable of informing food security initiatives in other contexts where seasonality is a limiting factor in food availability. A multitude of social, ecological, physiological and cognitive processes culminate in food culture through our dietary knowledge systems [68].

While a broad range of physical, political, and economic factors influence food choices, local values and morals are especially important. The availability of fish is of course necessary for the continued consumption of air-dried fish as food. All these factors are called into question when we examine the cultural conservation and persistence of air-dried fish as a common meal of symbolic value in the villages of the Faroe Islands. The presence of small-scale fishing (making it free food), local ecological conditions (making it possible to wind-dry), acquired taste-preferences, socio-cultural factors, and the importance of old-fashioned fish and meat dishes as key symbols of identity and belonging have contributed to the survival of *ræstur fiskur* [69, 70].

Despite the fact that there today are fast food restaurants in the capital Tórshavn, many Faroese children still appreciate traditional foods, including *ræstur fiskur*. As long as residents take pride in making home-made air-dried fermented fish, and as long as the demand for it remains, the food customs will continues to preserve in the local enculturation process. Thus, *ræstur* fish may well survive within the Faroese cuisine.

## Consent

Written informed consent was obtained from the child's parents for the publication of this report and any accompanying images.
